# Proposing a precision-enhancing method for sagittal plane alignment during total knee arthroplasty

**DOI:** 10.1186/s12891-025-08912-5

**Published:** 2025-07-09

**Authors:** Jisu Park, Hyeongyu Lim, Chong Bum Chang

**Affiliations:** 1 Department of Orthopaedic Surgery, SMG- SNU Boramae Medical Center, Seoul, Republic of Korea; 2 Department of Orthopedic Surgery, Nanoori Hospital, Suwon, Korea; 3 Department of Orthopaedic Surgery, Seoul National University College of Medicine, Seoul National University Bundang Hospital, 82, Gumi-ro 173 Beon-gil, Bundang-gu, Seongnam-si, Gyeonggi-do 13620 Republic of Korea

**Keywords:** Total knee arthroplasty, Alignment, Sagittal alignment, Planning method

## Abstract

**Background:**

Achieving appropriate alignment after total knee arthroplasty (TKA) is crucial for long-term survival of implant but alignment in sagittal plane is relatively not well studied. The purpose of this study was to (1) propose the preoperative sagittal planning method of TKA using X-ray, (2) validate its accuracy and (3) find factors contributing to inaccurate sagittal placement of the component.

**Methods:**

Ninety-three knees of 71 patients were prospectively reviewed. Preoperative sagittal planning for the femoral and tibial component was conducted using simple X-ray images. The postoperative X-ray taken six weeks after surgery was used to validate the proposed method. The angle between the reference line and the expected resection line before surgery was defined as the preoperative gamma angle for the femur and delta angle for the tibia. Postoperatively, the angle between the same reference line and the actual component was defined as the postoperative gamma and delta angles, respectively. The target angle range for the difference between the preoperative and postoperative gamma and delta angles was set as -2° to 2°. Demographic and radiologic factors between groups that fell within and outside the target angle range were compared.

**Results:**

Total 75 cases (80.6%) met the target angle range of distal femur resection. Femoral component tended to be placed in more flexed position than planned. Anterior femoral notching was not observed in all cases. Total 89 cases (95.7%) met the target angle range of proximal tibia resection. No factors were associated with the increased difference in preoperative and postoperative femoral and tibial prosthesis placement in multiple regression.

**Conclusions:**

The proposed sagittal planning method for TKA demonstrated an accuracy of 80.6% for the femoral component and 95.7% for the tibial component. Since this method does not require any programs and additional costs, it could be a good alternative in situations where robotic-assisted TKA is not available.

## Background

Achieving appropriate alignment after total knee arthroplasty (TKA) is crucial for long-term survival of implant [1, 2]. Many methods have been proposed to gain the desired alignment in coronal plane. However, the alignment of the prosthesis in the sagittal plane, especially for the femoral side, is not much studied and can easily be misplaced [3]. Femoral component placed in too much flexed position may cause limitation in extension, and the opposite case may cause anterior notching of femoral cortex [4]. Of these, femoral cortical notching should be avoided as it can lead to periprosthetic fracture [5, 6]. In conventional manual TKA, the sagittal alignment of femoral component is mainly decided by the position of intra-medullary (IM) guide. The entry point of femoral IM guide is usually set approximately 5 to 10 mm anterior to the top of intercondylar notch. However, there are individual variations in bowing of distal femur, and if not considered, it may result in undesirable sitting of the prosthesis [7–9]. Several studies have emphasized the importance of entry point [10–13]. Also, because the medullary canal of femur is wider in anterior-posterior plane, sagittal alignment is more vulnerable to errors than coronal alignment [14, 15]. However, despite these concerns that the femoral component might be misplaced in the sagittal plane, there has been no previous discussion focusing on methods to overcome this issue.

As tibial slope after TKA has an impact on biomechanics of the knee joint, it is also crucial to make an accurate tibial resection in sagittal plane [16, 17]. The sagittal mechanical axis of the tibia is typically represented by a line connecting the center of the tibial plateau to the center of the tibial plafond [18]. However, in patients with osteoarthritis, it can be challenging to clearly identify the center of the plateau on X-rays. In such cases, there are alternative reference lines: the fibular shaft axis (FSA), the anterior cortical line, the posterior cortical line, and the anatomical axis of the tibia. These landmarks all correlate well to the mechanical axis, and surgeons can choose whichever they are comfortable using with [18, 19]. For accurate tibial sagittal resection, choosing easy and reproducible landmark is important. This landmark should be easy to be recognized both in preoperative planning step and during intraoperative procedure. There are reports of using FSA for intraoperative reference because of its palpable nature [20, 21]. However, as FSA is not always palpable during the surgical procedure in cases like obese patients, FSA alone is not enough to create accurate tibial resection [22].

In this study, the authors propose a simple and reliable preoperative planning method to achieve appropriate and accurate sagittal placement of the prosthesis. Moreover, the factors that could influence the accuracy of sagittal alignment were evaluated. The purpose of this study was to (1) propose the preoperative sagittal planning method of TKA using X-ray, (2) validate its accuracy and (3) find factors contributing to inaccurate sagittal placement of the component. It was hypothesized that this method would achieve satisfying sagittal alignment and factors like bowing of bone, skin thickness and bone density could affect the accuracy.

## Methods

Ninety-three knees of 71 patients who underwent TKA for osteoarthritis between September 2023 and December 2023 were prospectively reviewed. Among 113 knees of 87 patients who consented for the study, 20 knees of 16 patients were excluded because of low quality of follow-up X-ray. A single surgeon planned the preoperative sagittal plane resection angle with knee lateral X-ray and lower leg lateral X-ray. Surgeries were performed by the same surgeon based on this method. Preoperative and postoperative X-ray were reviewed. This study was approved by the institutional review board of our hospital (IRB No: B-2308-845-305).

### Study subjects

Among patients scheduled to undergo primary TKA by a single experienced surgeon from September 2023 to December 2023, those with primary osteoarthritis were eligible for the study. Patients with rheumatoid arthritis, inflammatory arthritis, infective arthritis, and traumatic arthritis were excluded from this study. Also, patients with history of surgery or trauma to femur, tibia, or fibula were excluded. In case the appropriate X-ray was not achievable due to patients’ postural restriction, they were excluded. The demographic data of included cases are presented in Table 1.

**Table 1:** Demographic data of included cases

Age^*^ (year)		72.4 ± 6.3
Sex^†^		W: 76, M: 17
Height^*^ (cm)		155.8 ± 9.0
Weight^*^ (kg)		66.8 ± 11.6
BMI ^*^ (kg/m^2^)		27.4 ± 3.3
DXA^*^ (T-score)		−0.7 ± 1.1
Side^†^		R: 53, L: 40
Implant^†^	J2BCS	20
	Physica	26
	U2	34
	Exult	13

*W* women, *M* men, *BMI* body mass index, *DXA* dual-energy x-ray absorptiometry, *R* right, *L* left, *J2BCS* Journey II bi-cruciate stabilized

^*^The values are given as the mean ± standard deviation

^†^The values are given as the number of cases

### Implants and surgical procedure

Implants used in this study were Journey II bi-cruciate stabilized (J2BCS; Smith & Nephew, Memphis, TN, USA), Physica (Lima, Udine, Italy), U2 (United Orthopedic, New Taipei City, Taiwan), and Exult (Corentec, Seoul, Republic of Korea). Each surgery was performed using the instruments provided the manufacturer. In case of the tibial slope guide, J2BCS and Exult employed a 3° slope guide, while Physica and U2 used a 0° slope guide. In case of J2BCS and Exult, the tibia stem was tilted by 3° relative to the tibial plate in the sagittal plane. In order to avoid contact of tibial stem to posterior cortex of tibia, the provided 3° slope guide was used, resulting in J2BCS and Exult having a tibial slope that was 3° greater than planned. Therefore, for J2BCS and Exult, the validation of the postoperative angular difference was calculated with this additional 3° taken into account. The configuration characteristics of the implants are presented in Table 2. All surgeries were performed using a medial parapatellar approach, employing a modified gap technique, and aimed to achieve mechanical alignment. The prostheses used were posterior-stabilized (PS) type implants with a fixed bearing system, and fixation was achieved using cement.

**Table 2:** Configuration of the TKA implants

	Femur anterior flange angle (°)	Tibia sagittal stem angle (°)	Tibia slope guide (°)
J2BCS	3	3	3
Physica	5	0	0
U2	5	0	0
Exult	5	3	3

*J2BCS* Journey II bi-cruciate stabilized

### Preoperative planning

#### Femur

**Fig. 1:**
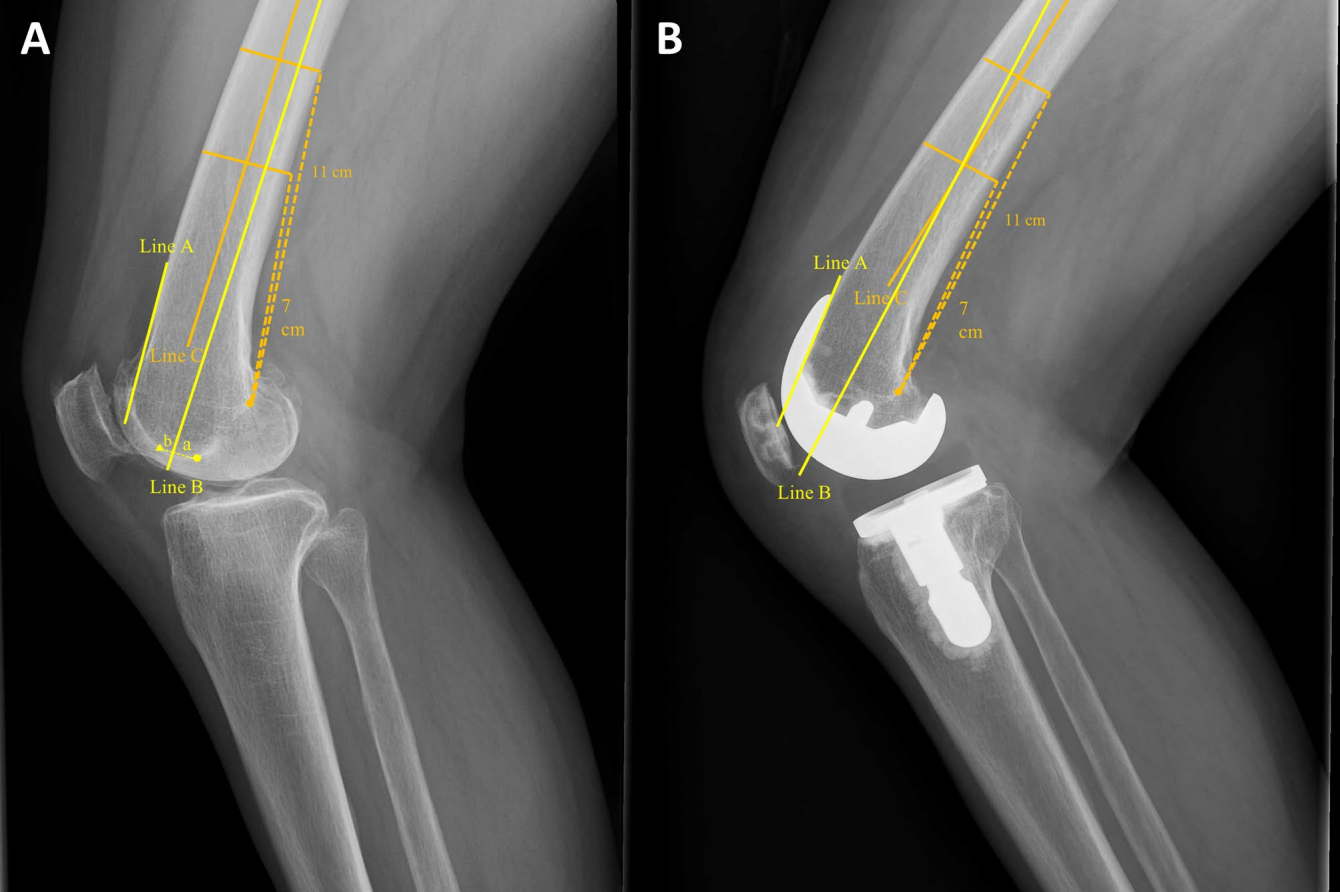
Planning and validation of the femoral prosthesis placement.** A** IM guide line (Line B) was drawn to be more flexed than the anterior femoral cortex (Line A) by the amount of anterior flange angle of the prosthesis. The distance from the intersection point of this line with the intercondylar groove to the most distal roof of the intercondylar notch (circle) was calculated (a). Half of the diameter of IM rod (b) was added to this distance (FED: a + b). The angle between the Line B and the reference line (Line C) was defined as preoperative gamma angle. **B** After TKA, Line B was drawn to be flexed than Line A by the amount of the anterior flange angle of the prosthesis. The angle between the Line B and C was defined as postoperative gamma angle

Planning was done considering the positioning of the femoral prosthesis in the sagittal plane. Knee lateral X-ray was used for the sagittal planning of the femur (Fig. 1A). To avoid anterior notching of the femur, the guideline was drawn taking into account the anterior cortex line of the femur and the anterior flange angle of the femoral component (Table 2). Accordingly, the path for IM guide (Fig. 1A, line B) was drawn in flexion by the amount of anterior flange angle relative to the anterior femoral cortex. This line was drawn to make contact with the posterior cortex of the medullary canal. The distance from the intersection point of this line with the intercondylar groove to the most distal roof of the intercondylar notch was calculated. Considering the diameter of IM rod, additional 4 mm were added to this distance (the used IM guide in this study was 8 mm in diameter and 350 mm in length). This was the final planned distance for IM rod entry point (femur entry distance: FED). Intraoperatively, the femoral IM rod insertion was made anterior to the intercondylar notch roof by FED along the Whiteside line (Fig. 1A, triangle).

#### Tibia

**Fig. 2:**
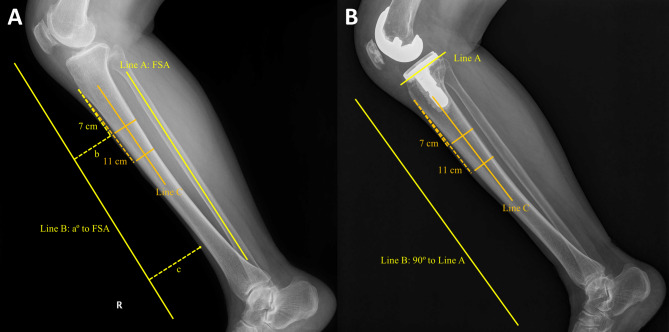
Planning and validation of the tibial prosthesis placement.** A** EM guide line (Line B) was drawn in front of the tibia, in extension by the amount of PSA (a) compared to FSA (Line A). The distances from two points to Line B was calculated (b, c). The difference of these two distances was noted, and EM guide was positioned in the operating field by using this value of difference (c-b). The angle between the Line B and reference line (Line C) was defined as preoperative delta angle. **B** After TKA, Line B was drawn tangential to the proximal tibia resection plane (Line A). The angle between Line B and C was defined as postoperative delta angle

Sagittal planning of the tibia was conducted using lower leg lateral X-ray (Fig. 2A). As all TKA prosthesis in this study were posterior-stabilized (PS) implants, to prevent excessively wide flexion gap after bone resection, the target posterior slope of tibia was set to be minimal, but intended to avoid reverse slope. Among several possible landmarks that can represent the sagittal axis of tibia, FSA was used in this study [19]. Basically, the posterior slope of tibia was set to be 0° to FSA. However, in case where patient had increased original tibial slope, there was a concern that the tibial component might impinge on the posterior cortex of tibia. To mitigate this risk, an additional angle was applied, resulting in the determination of the planning slope angle (PSA). Once the PSA was determined, extra-medullary (EM) guide line (Fig. 2A, Line B) was drawn in front of the tibia, in extension by the amount of PSA compared to FSA (increased PSA would result in increased posterior slope after resection). The distances from two points to EM guide line was calculated: first point was 7 cm distal along the anterior side of tibia from the tibia tuberosity; second point was the most concave anterior part around the ankle. The difference of two distances was noted, and EM guide was positioned in the operating field by using this value of difference.

### Validation of the planning method

For the validation of the method, knee lateral and lower leg lateral X-ray images obtained six weeks after surgery were used.

#### Femur: preoperative and postoperative gamma angle

Femur planning angle was measured at knee lateral X-ray (Fig. 1A). Two femur posterior cortex points were set 7 cm and 11 cm from the most posterior point of the Blumensaat line, and a line bisecting the outer cortical diameter of the femur at each point was set as the reference line (Fig. 1A, Line C). The angle between this reference line and planned IM guide line (Fig. 1A, Line B) was defined as preoperative gamma angle. The positive value represents relative flexion of planned distal resection plane.

After TKA, the real distal femur resection plane was measured by using anterior flange angle. The angle between line perpendicular to the distal femur resection plane (Fig. 1B, Line B) and the reference line (Fig. 1B, Line C) was defined as postoperative gamma angle.

#### Tibia: preoperative and postoperative delta angle

Tibia planning angle was measured at lower leg lateral X-ray (Fig. 2A). Two tibia anterior cortex points were set 7 cm and 11 cm distal from tibia tuberosity. A line bisecting the outer cortical diameter of tibia at each point was set as the reference line (Fig. 2A, Line C). The angle between this reference line and EM guide line (Fig. 2A, Line B) was defined as preoperative delta angle. The positive value represents increasing the posterior slope of tibia.

After TKA, the real proximal tibia resection plane was measured using the baseplate of tibia prosthesis (Fig. 2B, Line A). The angle between line perpendicular to the proximal tibia resection plane (Fig. 2B, Line B) and reference line (Fig. 2B, Line C) was defined as postoperative delta angle.

#### Target range angle

Preoperative gamma, delta angle and postoperative gamma, delta angle were compared respectively. The difference between preoperative and postoperative values was also calculated (gamma angle difference, delta angle difference). The target range was set as between − 2° to 2° of gamma and delta angle difference.

### Other radiological measurements

Before surgery, hip-knee-ankle (HKA) angle, TPS, and soft tissue thickness at 7 cm distal to the tibia tuberosity (tibia skin thickness: TST) were collected. The bowing of femur and tibia were measured by following methods. These measurements were used for the multiple regression analysis for risk factors of inaccurate sagittal placement of the prosthesis. The summary of radiologic measurements is presented in Table 3.

**Table 3:** Summary of radiologic measurements of included cases

HKA angle (°)	8.9 ± 3.7
DFB (°)	4.4 ± 1.7
PTB (°)	2.0 ± 1.5
TST (mm)	8.8 ± 3.8
TPS (°)	8.2 ± 4.3

The values are given as the mean ± standard deviation

*HKA* hip-knee-ankle, *DFB* distal femur bowing, *PTB* proximal tibial bowing, *TST* tibia soft tissue thickness, *TPS* tibia posterior slope

#### Distal femur bowing angle

**Fig. 3:**
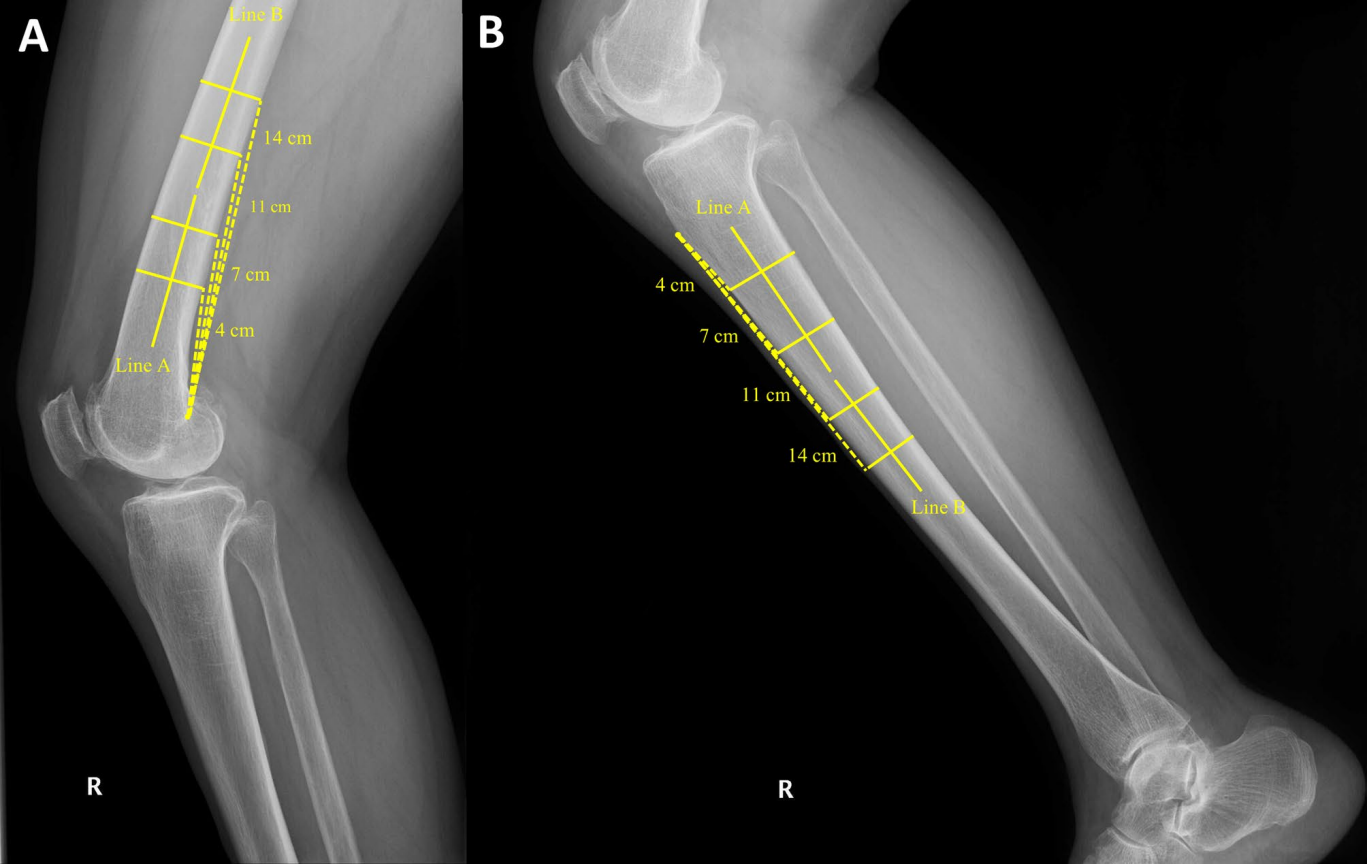
Measurements of bowing of femur and tibia.** A** The angle between Line A and B was defined as distal femur bowing angle (distal femur bowing: DFB). **B** The angle between Line A and B was defined as proximal tibia bowing angle (proximal tibia bowing: PTB)

Distal femur bowing angle was measured at knee lateral X-ray (Fig. 3A). Two line was drawn to calculate the distal bowing of femur. For the first line, two femur posterior cortex points were set 4 cm and 7 cm from the most posterior point of the Blumensaat line, and a line bisecting the outer cortical diameter of femur at each point was drawn. For the second line, two femur posterior cortex points were set 11 cm and 14 cm from the most posterior point of the Blumensaat line, and a line bisecting the outer cortical diameter of femur at each point was drawn. The angle between these two lines was defined as distal femur bowing angle (distal femur bowing: DFB). The positive value represents anterior bowing of distal femur.

#### Proximal tibia bowing angle

Proximal tibia bowing angle was measured at lower leg lateral X-ray (Fig. 3B). Two line was drawn to calculate the proximal bowing of tibia. For the first line, two tibia anterior cortex points were set 4 cm and 7 cm distal from tibia tuberosity. For the second line, two tibia anterior cortex points were set 11 cm and 14 cm distal from tibia tuberosity. Two lines bisecting the outer cortical diameter of the tibia at each point were drawn. The angle between these two lines was defined as proximal tibia bowing angle (proximal tibia bowing: PTB). The positive value represents anterior bowing of proximal tibia.

### Statistical analysis

A priori power calculation test was performed using G-power software (version 3.1.9.7; Kiel University, Germany). The sample size for a paired *t* test was calculated with a two-tailed effect size of 0.4, obtained from our previous pilot study, an alpha error of 0.05, and a power of 0.95. The calculated sample was 84 cases, and considering a dropout rate of approximately 25%, the study was designed to include 112 cases.

Descriptive statistical analysis was performed, and data normality was evaluated using Kolmogorov-Smirnov test. Continuous variables were compared using paired *t* test or the Wilcoxon Rank-Sum test. Multiple regression was performed to evaluate the risk factor of deviating from the target angle. The absolute values of the changes of gamma and delta angles were used for the multiple regression. All statistical analyses, except for the power analysis, were conducted using R (version 4.2.2; R Foundation for Statistical Computing, Vienna, Austria) and RStudio (version 2023.03.1 + 446; R Foundation for Statistical Computing, Vienna, Austria). Statistical significance was set as *P*-value < 0.05.

## Results

**Fig. 4:**
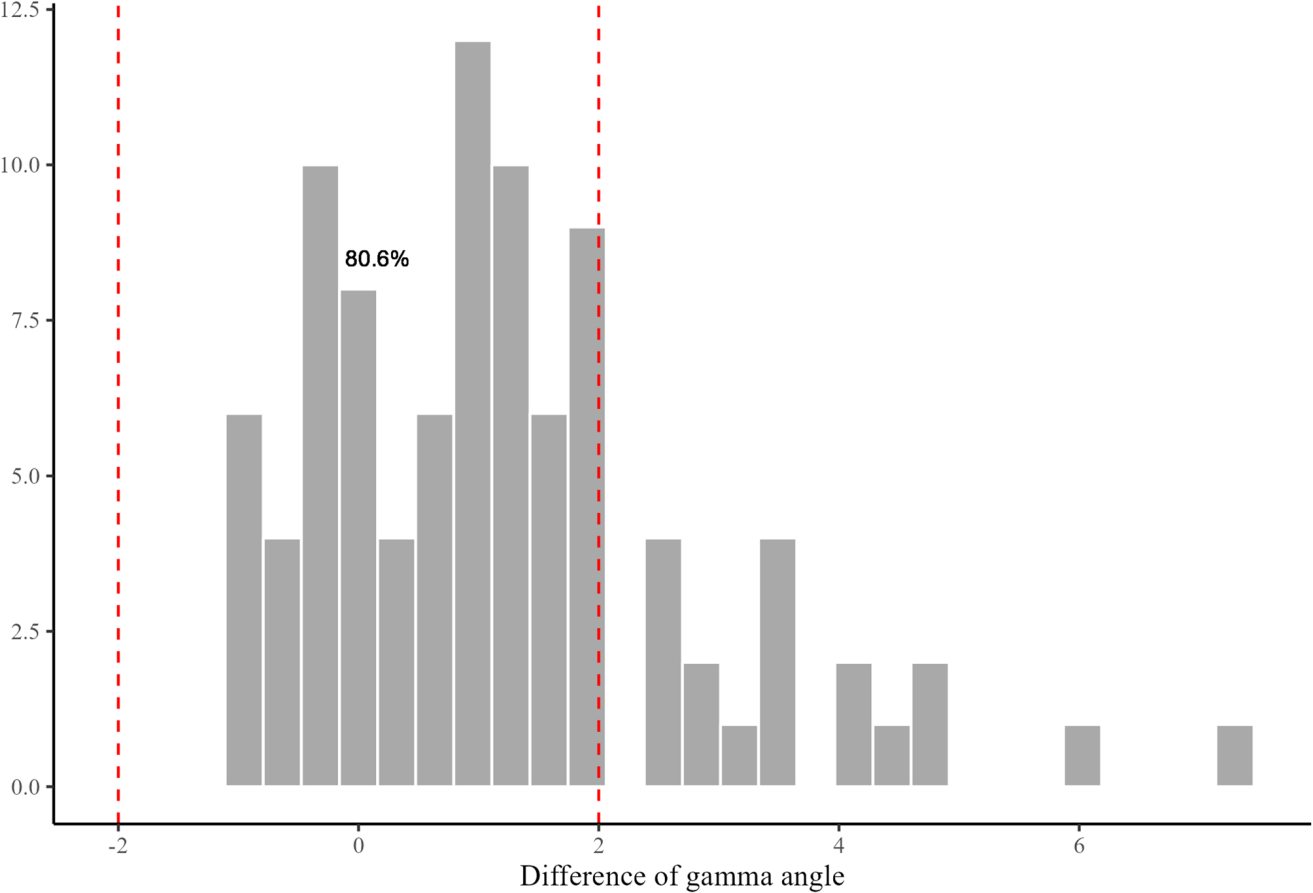
Difference between preoperative planning and postoperative femoral prosthesis placement. Total 80.6% cases were in the target range of ± 2° for the femoral component

**Fig. 5:**
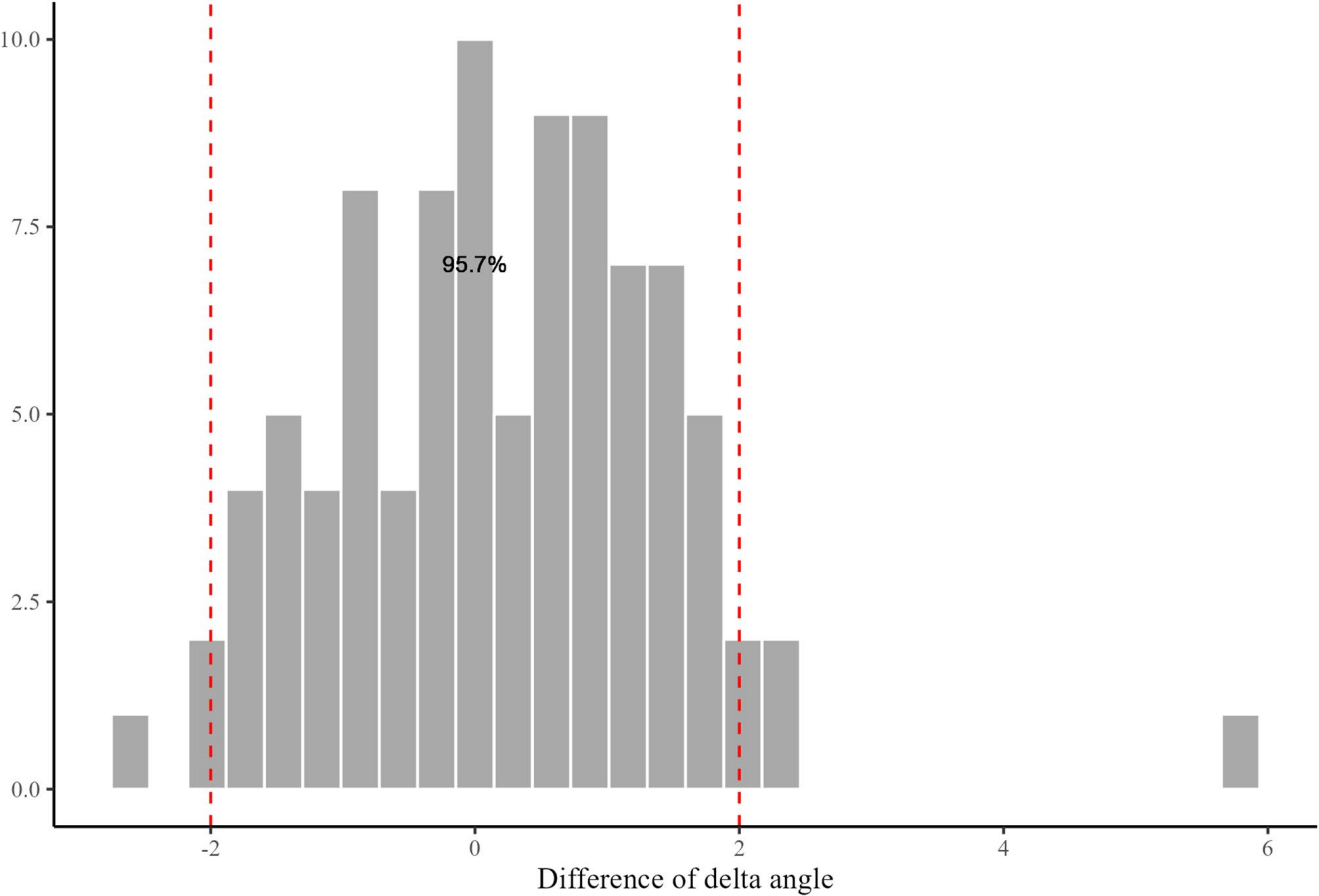
Difference between preoperative planning and postoperative tibial prosthesis placement. Total 95.7% cases were in the target range of ± 2° for the tibial component

The average postoperative gamma angle was 4.4°, which was greater than the preoperative gamma angle of 3.2° (Table 4). Total 75 cases (80.6%) met the target angle range of distal femur resection (Fig. 4). Anterior femoral notching was not observed in all cases. Postoperative delta angle was comparable to the preoperative delta angle (Table 4). Total 89 cases (95.7%) met the target angle range of proximal tibia resection (Fig. 5).

**Table 4:** Summary of planning and validation measurements

	Planning	Validation	*P*-value
FED^*^ (mm)	9.0 (0 to 20)	-	-
PSA^*^ (°)	0.8 (−1.0 to 4.0)	-	-
Gamma angle^†^ (°)	3.2 ± 1.9	4.4 ± 2.3	< 0.05
Delta angle^†^ (°)	2.5 ± 1.3	2.7 ± 1.4	0.153

*FED* femur entry distance, *PSA* planning slope angle

^*^The values are given as the mean with the range in parentheses

^†^The values are given as the mean ± standard deviation

Multiple regression analysis did not identify any factors that increased the gamma angle difference and delta angle difference (Tables 5 and 6).

**Table 5:** Multiple regression for gamma angle difference (femur)

Predictor	Unstandardized Coefficients		Standardized Coefficients	t	*P*-value	95% CI
	B	SE	Beta			
(Constant)	−3.729	2.335		−1.597	0.114	(−8.37, 0.91)
Sex (Men)	0.271	0.400	0.076	0.676	0.501	(−0.53, 1.07)
Age	0.040	0.025	0.182	1.603	0.113	(−0.01, 0.09)
BMI	0.080	0.048	0.189	1.652	0.102	(−0.02, 0.17)
DXA	−0.180	0.157	−0.139	−1.146	0.255	(−0.49, 0.13)
HKA angle	0.020	0.040	0.052	0.490	0.625	(−0.06, 0.10)
DFB	0.019	0.088	0.023	0.212	0.833	(−0.16, 0.20)

*BMI* body mass index, *DXA* dual-energy x-ray absorptiometry, *HKA* hip-knee-ankle, *DFB* distal femur bowing, *CI* confidence interval, *SE* standard error

**Table 6:** Multiple regression for delta angle difference (tibia)

Predictor	Unstandardized Coefficients		Standardized Coefficients	t	*P*-value	95% CI
	B	SE	Beta			
(Constant)	0.213	0.352		0.605	0.547	(−0.48, 0.9)
Sex (Men)	0.094	0.061	0.179	1.534	0.129	(−0.03, 0.21)
Age	−0.003	0.004	−0.084	−0.733	0.466	(−0.01, 0.0)
BMI	0.000	0.007	−0.003	−0.026	0.979	(−0.01, 0.01)
DXA	−0.016	0.024	−0.086	−0.681	0.498	(−0.06, 0.03)
HKA angle	−0.005	0.006	−0.095	−0.868	0.388	(−0.02, 0.01)
TPS	−0.003	0.005	−0.061	−0.537	0.593	(−0.01, 0.01)
TST	−0.003	0.006	−0.053	−0.460	0.647	(−0.01, 0.01)
PTB	0.003	0.015	−0.019	0.177	0.860	(−0.03, 0.03)

*BMI* body mass index, *DXA* dual-energy x-ray absorptiometry, *HKA* hip-knee-ankle, *TPS* tibia posterior slope, *TST* tibia soft tissue thickness, *PTB* proximal tibial bowing, *CI* confidence interval, *SE* standard error

## Discussion

In this study, new planning method to achieve appropriate TKA sagittal alignment based on simple X-ray images was proposed. The primary finding of this study was that the desired postoperative sagittal alignment could be achieved using the proposed sagittal planning method. The accuracy was higher in tibia, and the femoral component showed tendency of being placed relatively more flexed than preoperative planning. No risk factors to misplace the femoral and tibial component in sagittal plane were found.

The accuracy of sagittal placement of femoral component was 80.6%. Although there is limited research on the sagittal placement of the femoral prosthesis, no study has yet investigated methods for achieving proper sagittal alignment. In cadaver study by Chauhan et al., appropriate sagittal alignment was more difficult to achieve compared to coronal alignment [23]. Pfitzner et al. have showed the technique of controlling IM guide for femoral resection, but only for the coronal alignment [24]. According to study by Haruta et al., there could be a deviation of the IM rod up to 2° depending on whether the proximal end of the rod touched the anterior or posterior cortex [15]. To avoid this error, the proposed planning in this study was targeted to touch posterior cortex of femoral canal in preoperative planning step, and the IM rod was inserted in the same manner during surgery. Mihalko et al. have showed that alignment can be significantly affected by the starting point of IM guide in their cadaveric study [11]. There are several other 3-dimentional image-based studies proposing the importance of femoral entry point in placing the IM guide [13, 25]. In this study, the femoral entry point was 9.0 mm in average, ranging from 0 to 20 mm anterior to the top of the intercondylar notch (Table 4). If the femoral IM guide entry point had been uniformly fixed at either 5–10 mm, notching might have occurred in some cases. Since the anterior bowing of the femur varies by patients, it would be reasonable to adjust the entry point on a case-by-case basis. Although the overall accuracy of femoral component placement was 80.6%, there was a tendency for the femoral component to be placed approximately 1.2° more flexed than planned. Interestingly, there were no cases where the femoral prosthesis was inserted with more than 2° of extension compared to the plan. All outliers were in the direction of flexion, with a maximum difference of up to 7.2°. This flexion tendency has also been reported from other studies. In study by Mancino et al., comparing robotic-assisted TKA (RA-TKA) with navigated TKA (NA-TKA), femoral component was placed 0.9° flexion from the target in RA-TKA and 1.9° flexion from the target in NA-TKA [26]. Also, in study regarding the accuracy of RA-TKA by Rossi et al., there was a statistically significant difference in planning and validation of femoral component sagittal angle by 0.8° [27]. Femoral component seems easily to be placed in flexion than planned even using RA-TKA. However, according to the study by Farooq et al., in which machine learning algorithm was used to identify the optimal sagittal component position in TKA, increased likelihood of favorable outcome was reported when the femoral component was positioned between 0° and 7° flexion [28]. In their study, worse outcomes were predicted with femoral component extension. In computer simulation study by Nishitani et al., femoral component placed more than 10° flexion caused paradoxical anterior translation of the medial compartment [29]. According to their another clinical study, sagittal alignment of the femoral component more than 8.5° showed inferior clinical outcomes [30]. While the observed tendency toward increased femoral component flexion in our study appears to remain within the clinically acceptable margin, the possibility of excessive flexion necessitates careful attention. Given that the femoral component was placed approximately 1.2° more flexed than planned, one practical consideration may be to intentionally plan for 1° more extension during the preoperative planning step.

More than 95% were within the desired target angle range in case of tibial component. Several methods have been proposed to make an accurate and appropriate proximal tibia resection. Some advocates the accuracy of IM guide for tibial resection [31]. It is an easy and convenient method but has the potential being inserted inappropriately when tibia has bowing or in case when medullary canal is narrow or wide [32]. Using EM guide for tibia resection can be free from these concerns, but requires additional landmarks to ensure proper alignment. Among many landmarks for tibial sagittal alignment, FSA was used in this study, as it is a simple and reproducible landmark which correlates well to mechanical axis of tibia [18]. While it is also possible to use FSA as a reference line during surgery by palpating the fibula, this approach is not feasible in all cases, such as with obese patients. Therefore, we calculated the distance from imaginary EM guideline to skin at two points in planning step and used this value during operation. There are several other studies demonstrating similar methods. Tsukeoka et al. calculated the similar distance using CT scan image and reported 89.1% of accuracy within 2° target [33]. Kim et al. measured distance using X-ray and by using tibia anatomical axis as reference line [22]. They reported 78.7% of accuracy which was higher compared to intraoperatively palpating fibula method. In this study, by using the FSA as the planning reference and employing the method of measuring the distance from the EM guide to the tibia skin during surgery, a high level of accuracy could be achieved. The method proposed in this study is encouraging as this level of accuracy was achieved by using simple, routinely taken X-rays and straightforward planning.

No risk factors for the misplacement of the femoral and tibial components in the sagittal plane were identified. Given that femoral bowing can influence the insertion of IM guide, it was originally hypothesized that this could be a risk factor for misplacement. Hood et al. posed the possibility of femoral component being placed relatively hyperextension in patients with increased femoral bowing [34]. Ko et al. also showed the risk of anterior notching of femur in NA-TKA when femur had increased bowing [9]. However, in this study, anterior notching was not observed, and femoral bowing was not found to contribute to misplacement of the femoral component. This is believed to be due to the fact that bowing was taken into account during the planning stage. Additionally, thickness of the tibial skin was considered as a potential risk factor as it can interfere intraoperative placement of EM guide for tibia, but not found to be relevant. This is also thought not to have affected the outcomes since it was already considered during the preoperative planning stage. Furthermore, low bone density was not related to the outcome. It seems that proposed method can be utilized regardless of the demographic characteristics of patients, though some execution errors should still be considered.

Recently, RA-TKA is emerging. The proportion has been increasing annually over the past decade, accounting for 5.89% of all TKAs in the United States in 2020 [35]. One of the most important advantages of using robot is accurate bone resection. There are increasing number of studies showing that RA-TKA had better postoperative alignment compared to the conventional manual method in coronal plane [36, 37]. In the sagittal plane, however, the superiority of RA-TKA is difficult to determine. Although robot allows accurate resection, if adequate reference line is not used, the following alignment can be far from expectation. An et al. compared the sagittal alignment of seven different brands of robotic-assisted TKA systems [38]. Interestingly, all seven systems used the same sagittal reference for femoral side, but tibial axis was different by brands. In case of femoral prosthesis, mechanical axis of femur is set as the line connecting the center of femoral head and knee in RA-TKA. This line is used as a reference line of distal femoral resection both in coronal and sagittal plane [39]. In case of conventional manual TKA, distal femoral resection is done by using the inserted IM guide. According to literature, the mechanical axis used in RA-TKA is usually in more extended position compared to manual axis, with an angular discrepancy of 2.46° to 3.8° [39, 40]. Because of this discrepancy, it is unreasonable to conclude that RA-TKA is more accurate by evaluating with the use of the axis of RA-TKA. It is also worth noting that most current implants are designed for traditional intramedullary guidance, so the robot may actually end up placing the femoral component in more extended position than expected. Furthermore, RA-TKA is not feasible to all patients. Not all institutions have the robot and not all patients are affordable of the additional costs [41]. The proposed method in this study is simple, doesn’t take much time, and doesn’t require any additional costs. In situations where robotic surgery is not available, this method has the advantage of showing fairly accurate and predictable results.

There are several limitations to this study. First, the result is only about the accuracy of preoperative planning. The implant was able to be positioned as planned in the sagittal plane, but this study did not address the outcomes related to sagittal alignment. Determining the optimal sagittal alignment for the femoral and tibial components falls outside the scope of this paper. For example, some surgeons might consider a 0° slope relative to the tibial axis appropriate, while others may prefer a 3° slope. However, this paper is not focused on identifying the ideal angle; rather, its objective is to present a planning method that can reliably reproduce any chosen value. Second, there is no the inter-, intra-observer reliability data available. The absence of multiple evaluators is a limitation regarding the study’s validity. However, since relatively clear landmarks were used, inter-, intra-observer variation is expected to be minimal. Third, the comparison with RA-TKA was not conducted. Although RA-TKA was not available at the institution where the study was conducted, precluding a direct comparison, the results still demonstrated a satisfactory level of accuracy. Future validation through comparisons with RA-TKA is warranted.

## Conclusions

The proposed sagittal planning method for TKA demonstrated an accuracy of 80.6% for the femoral component and 95.7% for the tibial component. Since this method does not require any programs and additional costs, it could be a good alternative in situations where robotic-assisted TKA is not available.

## Data Availability

The datasets used and/or analyzed during the current study are available from the corresponding author on reasonable request.

## Abbreviations

TKA: Total knee arthroplasty
FSA: Fibular shaft axis
IM: Intra-medullary
EM: Extra-medullary
PS: Posterior-stabilized
FED: Femur entry distance
TPS: Tibial posterior slope
PSA: Planning slope angle
HKA: Hip-knee-ankle
TST: Tibia skin thickness
DFB: Distal femur bowing
PTB: Proximal tibia bowing
RA-TKA: Robotic-assisted total knee arthroplasty
NA-TKA: Navigated total knee arthroplasty
